# Socialized to (Dis)trust? A Panel Study into the Origins of Dispositional Institutional Trust

**DOI:** 10.1007/s11205-025-03564-3

**Published:** 2025-04-10

**Authors:** Chaïm la Roi, Carmen van Alebeek, Tom van der Meer

**Affiliations:** 1 Planbureau Fryslân, Leeuwarden, The Netherlands; 2 Department of Political Science, University of Amsterdam, Amsterdam, The Netherlands

**Keywords:** Institutional trust, Socialization, Dispositions

## Abstract

A longstanding argument in the field of institutional trust reads that trust is the outcome of a process of socialization. This approach suggests that institutional trust may be understood as a set disposition that is shaped during one’s impressionable years (i.e., adolescence and pre-adulthood) and no longer systematically updated during an iterative process afterwards. Consequently, this disposition forms a baseline around which trust judgments tend to vary. Yet, this process of socialization to (dis)trust has not been studied directly. To fill this gap, this paper tests two rivalling models derived from cultural sociology. The active updating model implies that attitude baselines continue to be updated durably throughout a lifetime, whereas the settled dispositions model suggests that these attitudes remain relatively stable over a lifetime: longitudinal variation can be understood as random noise to the model. To test these models, this paper employs two panel data sets in the Netherlands (2018–2022) that measure trust in politics and other institutions annually: the LISS panel (covering the adult population) and the Dutch Adolescent Panel on Democratic Values (covering students in secondary education from age 12). We find evidence supporting the impressionable years hypothesis: while political trust is still subject to repeated updating among adolescents, it has settled into a disposition among adults. As such, our study highlights the relevance of socialization processes for the formation of institutional trust (during adolescence), as well as the relevance of a dispositional root of public attitudes (during adulthood). These findings have important implications for our understanding of both the determinants and consequences of institutional trust.

## Introduction

Against the backdrop of decreasing levels of trust in public institutions in established democracies (Zmerli, 2012), the consequences of institutional trust have garnered considerable scholarly attention in recent decades. As institutional trust is often regarded as an indicator of public support for the primary institutions upholding modern democratic societies (e.g., judicial, political, educational and policing institutions), many have investigated the potential consequences of (dis)trust in public institutions for various outcomes, including democratic participation (Levi & Stoker, 2000; Brehm & Rahn, 1997; Hooghe & Marien, 2013), compliance (Scholz & Lubell, 1998; Feld & Frey, 2007; Marien & Hooghe, 2011), and even support for democratic principles (Easton, 1965; Norris, 1999).

Despite this focus on the observed changes in trust patterns and their implications, much remains unclear about their durability. This stems from the tendency of the literature on the consequences of institutional trust to steer clear of formulating concrete expectations about how long these shifts can be anticipated to last. Yet, in order to comprehend whether observed macro-level changes in institutional trust reflect deeply rooted cultural trends or temporary phenomena, it is crucial to understand the origins of institutional trust as a public attitude. In other words, it is impossible to fully understand the long-term implications of trust changes without considering how institutional trust is generated and evolves within individuals over time. As a result, scholars in this field have often found themselves engaged in an ongoing theoretical debate regarding the degree of stability and endurance inherent in citizens’ institutional trust.

This debate has given rise to two leading perspectives on the nature of institutional trust. On the one hand, some argue that trust in public institutions is highly malleable and volatile, as it is presumed to be updated in response to institutional performance and events (e.g., Bowler & Karp, 2004; Chanley et al., 2000; Hetherington, 1998; Ouattara et al., 2023). An alternative approach contends that institutional trust is the result of an extensive process of socialization, portraying trust as enduring and relatively stable over a lifetime (e.g., Almond & Verba, 1963). This notion of trust as a stable disposition forms a prerequisite for theories that explain changing levels of institutional trust in Western European democracies as the outcome of generational replacement and cohort effects (Inglehart, 1997; Putnam, 2000; Dalton, 2004).

The latter approach presumes that institutional trust contains a strong dispositional element that remains constant after it has been formed during individuals’ early years of socialization (Jennings & Niemi, 1974). This suggests that individuals develop a lasting baseline of (dis)trust based on their experiences and influences in the initial stages of political and social learning during adolescence and young adulthood. Once formed, this baseline is set and no longer systematically updated in an iterative process.

However, this process of socialization to (dis)trust has not been studied directly. Many studies have examined the impact of important socializing agents on the shaping of levels of institutional trust, such as schools (e.g., Jennings & Niemi, 1974; Hooghe et al., 2015; Claes & Hooghe, 2017) or families (e.g., Jennings et al., 2009; Jennings, 2002). Moreover, more recent work has focused on the degree of stability of institutional trust in adults (Devine & Valgarðsson, 2024). Yet, a direct and systematic comparison of the development of institutional trust during formative and adult years within the same institutional and temporal context has been missing in the field. Moreover, extant studies have not directly assessed rivalling models to describe the structure of trust attitudes. This paper seeks to fill this gap by conducting a more direct test to determine to what extent institutional trust is structured by a stable disposition, and – if this is the case – at which age and under which conditions citizens develop this disposition.

In pursuit of this goal, we assess two rivalling models originating from cultural sociology on the formation and longevity of public attitudes: the active updating model (AUM) and the settled dispositions model (SDM) (Kiley & Vaisey, 2020; Vaisey & Kiley, 2021). According to the former, people continue to update their attitudes throughout their lives, making durable within-person changes without an underlying disposition that stabilizes these changes. The SDM suggests that these attitudes are relatively stable in the long run, as people have settled into a disposition to trust or distrust. In this model, trust continues to be open to longitudinal variation around this disposition, for instance as the consequence of short-term evaluations or non-attitudes, but these variations revert to the disposition over time. We therefore argue that the models of socialization and evaluation may be combined. The possibility of trust dispositions does not preclude the influence of short-term evaluations.

The socialization thesis predicts that people are not born with trust dispositions, but that they are developed over time, particularly during the ‘impressionable years’ of adolescence and young adulthood. Hence, we expect the AUM to dominate at a young age. If dispositions to trust or distrust exist, we would expect that institutional trust has settled into such a disposition among adults. Hence, the SDM should more effectively describe trust dynamics during adulthood.

To test these models, this paper employs two longitudinal data sets in the Netherlands (2018–2022): the LISS panel (covering the adult population) and the Dutch Adolescent Panel on Democratic Values (covering students in secondary education from age 12). Using structural equation modelling (SEM), we show that institutional trust forms a settled disposition for adults, while it is still subject to repeated updating among adolescents. Our main argument is therefore that institutional trust has a dispositional element that is shaped during one’s impressionable years but no longer systematically updated during an iterative process afterwards. Among adults this disposition forms a baseline around which trust judgments tend to vary over time. As such, this study emphasizes the relevance of socialization processes for the formation of institutional trust during adolescence as well as the importance of a dispositional root of trust during adulthood. These findings are vital for our understanding of both the determinants and implications of institutional trust.

## Theory and Hypotheses

### Institutional Trust as a Disposition

As a relational concept, trust can be conceptualized in terms of the actors that it involves: ‘subject A trusts object B to do X’ (Hardin, 2000). In the case of institutional trust, ‘subject A’ refers to citizens expressing their trust in public institutions (‘object B’). Consequently, different theories of institutional trust formation emphasize different elements of this conceptual sequence. The dispositional approach highlights the role of the trust subject A in the formation of institutional trust. However, it is in no way incompatible with the evaluative approach that emphasizes the influence of the traits of object B (fairness, output) on levels of trust. Dispositions may function as a baseline around which trust levels fluctuate and differentiate (cf. Zmerli & Newton, 2017), or as a benchmark against which the object of trust is evaluated.^1^

The dispositional argument posits that institutional trust judgments are primarily influenced by the trustor’s own characteristics or dispositions, and not by external stimuli such as enacted policies (Hetherington, 1998), election results (Anderson & LoTempio, 2002), or scandals (Bowler & Karp, 2004; Mishler & Rose, 2001). Because this disposition can be understood as a stable baseline of (dis)trust, it is presumed to be a lasting framework throughout one’s lifetime. Accordingly, any substantial changes or inequalities in trust in public institutions must reflect structural differences in the way these personal dispositions are shaped.

This shaping of dispositions is assumed to take place through socialization. As a core component of social learning theory, socialization refers to the process through which individuals acquire political and social beliefs, values, and attitudes (Greenberg, 1970). According to social learning theory, individuals form their values, attitudes, and behaviors through interactions with their immediate environment, adopting the values, attitudes and behaviors endorsed by their community (Marczewska-Rytko, 2020). Central to this idea is the influence exerted by various social agents, including family, peers, schools, and media.

While some argue that socialization processes persist into adulthood (e.g., Hyman, 1959; Niemi & Sobieszek, 1977), a widely shared consensus maintains that the majority of socialization occurs during individuals’ formative or ‘impressionable’ years (Inglehart, 1997). This period, typically encompassing adolescence and early adulthood, is characterized by heightened receptivity to external influences and a greater propensity for individuals to internalize societal norms, values, and behavioral patterns. Hence, we anticipate that individuals’ political and societal dispositions are subject to continuous updating during adolescence and young adulthood, but that they have largely solidified once these formative years have passed.

Several mechanisms contribute to this process of institutional socialization. One argument reads that individuals imitate the actions and attitudes of those they consider social role models (Bandura, 1971). If these social models display (dis)trust towards public institutions, it is likely that the learning recipient will adopt a similarly (dis)trustful stance. Conversely, it is also argued that individuals’ attitudes vis-à-vis public institutions is shaped by their first direct encounters and interactions with these institutions (Almond & Powell, 1978). However, since many adolescents are unlikely to have first-hand experience with public institutions – particularly political institutions – schools are often viewed as their first institutional encounter (Dewey, 1926; Claes et al., 2012). Here, it is presumed that pupils’ interactions with educational authority figures will have a lasting impact on their confidence in other public authorities. If these experiences are predominantly negative (positive), we can expect these individuals to develop a distrusting (trusting) predisposition towards public institutions.

The hypothesis that political orientations remain stable after the impressionable years has received mostly indirect and partial empirical evidence over the years. The most direct evidence is provided by Devine and Valgarðsson (2024) on political trust. They find that while trust “may be quite malleable in individuals’ formative years” (i.e., among young adults aged 16 to 18), it tends to remain stable in the long term throughout adulthood. Yet, perhaps somewhat paradoxically, short-term stability is not the defining factor that proves the existence of stable dispositions (Vaisey & Kiley, 2021). Rather, stable dispositions reveal themselves in the stability underlying short-term changes, whereas the existence of socialization is evidenced by an ongoing process of active updating (Kiley & Vaisey, 2020). In other words, to assess the dispositional structure of institutional trust, we need to assess changes upon changes.

Other studies offer less conclusive evidence for the impressionable years hypothesis. For instance, Claes et al. (2012) show that trust in political institutions is relatively stable between the age of 16–18 and two years later. A similar message is conveyed by Alwin and Krosnick’s (1991) earlier finding, using samples of adults (18 and above), that political attitudes do not get increasingly stable as individuals get older after the age of 18. Employing panel data from Belgian students between ages 16 and 21, Hooghe et al. (2015) demonstrate that differences in political trust between those who attended higher education and those who did not already manifest themselves at age 16. This implies that such inequalities are likely to result from preadult socialization experiences, and persist into adulthood. These studies thus reveal relative stability in later adolescence, although the onset of this stability is not assessed directly. They do not offer conclusive evidence for the impressionable years hypothesis, however. Rather, these studies examine the stability of political orientations either during adolescence or adulthood, lacking systematic comparisons across significant life stages.

More comprehensive tests of the impressionable years hypotheses are found in neighbouring fields of study. Jennings (2002) shows that the gap in various political orientations between groups of protesting and non-protesting students within the same generation also existed among their parents, while intragenerational stability over time proves to be high after young adulthood. Similarly, using a monetary trust game, Sutter and Kocher (2007) observe that trust in other people is shaped during childhood and adolescent years, and remains constant after adulthood.

### Two Models of Public Attitude Development, and Their Integration

Roughly parallel to this dispositional view of trust in public institutions as the product of early socialization are two models of public attitude change derived from cultural sociology: the active updating model (AUM) and the settled dispositions model (SDM) (Kiley & Vaisey, 2020; Vaisey & Kiley, 2021). The AUM assumes that individuals’ public attitudes are continuously and durably updated in reaction to new information and external stimuli. In the case of institutional trust, such stimuli or ‘shocks’ can include specific events, scandals or institutional output. This model implies that the development of institutional trust within individuals over time resembles an iterative process and shows a very low degree of stability. In other words, the model lacks a dispositional element, but makes sense at a stage when dispositions are formed because (young) people “adapt their views and make new meaning” (Kiley & Vaisey, 2020: 478).

On the other hand, the SDM posits that public attitudes reflect a dispositional element that is relatively stable from the moment that it is shaped early on in life. According to this model, attitudes constitute a consistent baseline that displays much greater resistance to substantial change. From the perception of the model, short-term changes in one’s attitudes over time tend to regress to the mean (i.e., the disposition) over time. Or, in the words of the original authors: “The SDM thus allows for population-wide shifts in beliefs, practices, or identities at a particular time (temporary period effects), but it assumes that within individuals these shifts will be erased over time as people return to their baselines” (Kiley & Vaisey, 2020: 481). The SDM does not presuppose perfectly stable attitudes, but long-term stability underlying short-term fluctuations. It can thereby account for both short-term, reactive evaluations (cf. Ouattara et al., 2023) and the randomness of non-attitudes (cf. Zaller, 1992). Yet, the long-term stability underlying possible short-term fluctuations is key to indicate dispositions.

The generation of most public orientations follows the SDM among adult citizens (Vaisey & Kiley, 2021). In fact, out of all attitudes included in their wide analysis of 183 variables in the General Social Survey, trust judgments show the highest likelihood of settling into a stable disposition. Factors such as behaviors, habits, or possessions are more likely to undergo active and lasting updating.

Yet, the two models can be combined. Even stable dispositions need to crystallize. We argue that the prevalence of the two models is likely to differ with age, so that AUM dominates when people are socialized into society as democratic citizens (e.g., at young age) whereas SDM dominates once people are settled in their roles (e.g., in adulthood). Kiley and Vaisey (2020) conclude that “the persistent change we *do* see in the data is somewhat more concentrated among younger respondents. On several items, it appears younger adults are still in the process of acquiring dispositions and habits they will take into later life” (p. 478). Their findings suggest attitudes may stabilize after they are crystallized into a set disposition at the end of one’s impressionable years, in a process that Kiley and Vaisey describe as ‘early acculturation’. This implies that AUM may dominate during the impressionable years, whereas SDM dominates with the onset of adulthood.

A recent comment on this argument suggests that the potential for persistent attitude change among adults may be greater than the SDM assumes (Lersch, 2023). Transformative life events such as parenthood, divorce or changes in employment, have been shown to shift individuals’ attitude baselines for an extended period of time. While such events may induce shifts in attitude baselines during adulthood, these impacts tend to be modest, and the durability of their influence remains uncertain. Consequently, these shifts are likely to be less impactful and less frequent than those during the impressionable years, when attitudes are still in the process of formation and adaptation.

All in all, building on social learning theory and indirect empirical evidence, we formulate our central hypothesis. It entails that institutional trust is shaped through active updating among adolescents, who are in the process of assigning meaning to (their position in) public life. Yet, once these ideas solidify, we expect to find evidence that institutional trust is in part settled among adults. The dominance of each model is thus conditional on age.

#### H1

Institutional trust follows the SDM in adults, but AUM in adolescents. (Impressionable years hypothesis).

We formulate two contrarian positions against our core hypothesis. Starting from a more purist notion that institutional trust is a stable disposition, a rivalling hypothesis reads that the development of institutional trust over time follows the SDM, even during the formative years of adolescence. In contrast to the socialization thesis, this presupposes the absence of a learning curve during adolescence and early adulthood, because trust is more deeply rooted in the subject (putting more emphasis on the ‘A’ in Hardin’s equation). There are substantive reasons why this is a viable alternative argument. The roots of the disposition may go further back than the formative years. For instance, the trust literature suggests that trust may be associated with genetics or stable psychological factors (Mondak et al., 2017). Alternatively, institutional trust has also been perceived as a non-attitude at all stages of life (Zaller, 1992).

#### H2

Institutional trust follows the SDM in both adolescents and adults. (Stable disposition hypothesis).

The final rivalling hypothesis states that individuals’ institutional trust fails to adopt a lifelong stable disposition but rather maintains flexibility throughout a lifetime. In other words, the AUM would dominate throughout the life course. Two potential mechanisms underpin this expectation. First, assuming the highest level of volatility, the evaluative approach to the formation of institutional trust suggests that the orientation responds mainly to external stimuli. The premise here is that individuals continuously reassess their confidence in public institutions by evaluating institutional performance against their own normative expectations (Miller & Listhaug, 1990; Bowler & Karp, 2004). In this scenario, citizens never establish a baseline for institutional trust, but merely react to changes at the institutional level.^2^ This model suggests that trust is purely reactive and not underpinned by an internal norm or benchmark (cf. Ouattara et al., 2023).

Second, it may also be the case that the formative years do result in a baseline of trust, but that this baseline changes over the course of a lifetime (Sigel, 1989). This is in line with the concept of ‘secondary socialization’, which assumes that socialization continues after the impressionable years (i.e., primary socialization) (Berger & Luckmann, 1966). In contrast to primary socialization, secondary socialization occurs as individuals adjust their initial beliefs in the long term, influenced by new interactions and experiences with institutions (Damico et al., 2000; Dahlberg & Linde, 2018). While such lasting ‘shocks’ to established attitudes and orientations are mainly presumed to be invoked by institutional performance (such as institutional failures or even events such as war, economic crisis, or a pandemic that may drive not only a short-term rally effect but can also impose long-term reorientations), they may also result from changes in individuals’ personal environment, such as the workplace (Geurkink et al., 2024). Taken together, both mechanisms lead to the same hypothesis that institutional trust is subject to regular updating among both adolescents and adults:

#### H3

Institutional trust follows the AUM in both adolescents and adults. (Active updating hypothesis).

## Data and Methods

### AUM and SDM

To distinguish between the two rivalling models, we build on the approach put forward by Vaisey and Kiley (2021). They argue and show that structural equation modelling (SEM) allows us to model both the AUM and SDM models and to compare which of the two types of models fit the data best. The main advantage of the approach Vaisey and Kiley used in 2021 over the one they used 2020 (see Kiley & Vaisey, 2020) is that the SEM model employs the fit of the model on all observed data waves, and not merely that of wave 3. In other words, the SEM model tells us how well the AUM and SDM models fit respondents’ complete response patterns across all waves, not just their responses in the final wave.

**Fig. 1:**
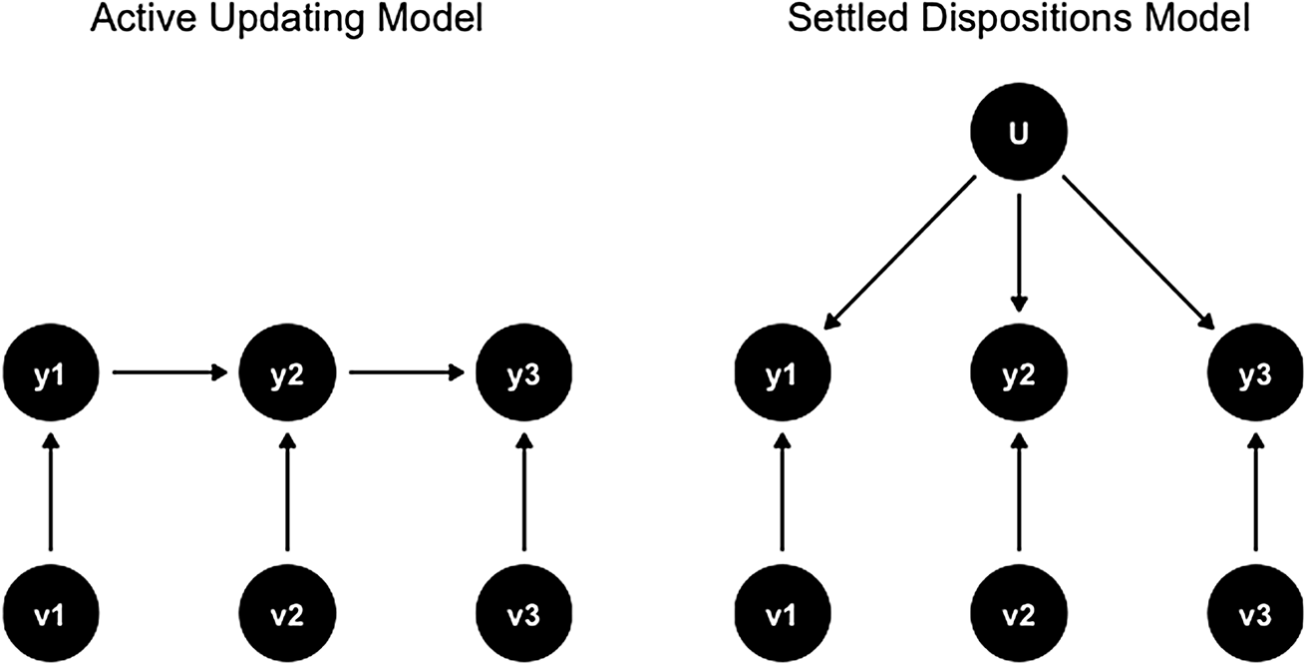
AUM and SDM models. (source: Vaisey & Kiley, 2021)

Vaisey and Kiley (2021) have graphically summarized the two rivalling models along the scheme provided in Fig. 1. In essence, the AUM argues that changes in opinions in earlier waves affect opinions in later waves. These changes are thus persistent. The pure AUM has no need for an underlying disposition that binds answers in different waves together. By contrast, the SDM at its core assumes that answers in different waves are driven by an underlying disposition (*U*). In the pure SDM, variations around that disposition may exist, but average to zero in the long run. In other words, “settled opinions does not necessarily mean stable (unchanging) opinions. People might change their responses from wave to wave, but the settled dispositions model assumes this change does not tend to persist, meaning it reflects temporary changes in opinion or measurement error” (Vaisey & Kiley, 2021: 90–91).

To distinguish between the two main models, Vaisey and Kiley (2021) transposed them to a SEM format. The test of rivalling models requires panel data with at least three waves. We follow the design by Kiley and Vaisey closely, and therefore stick to the setup of three-wave panel data. Modelling four consecutive waves could have been interesting, but this would have led to a significant loss of cases due to imperfect panel participation.

**Fig. 2:**
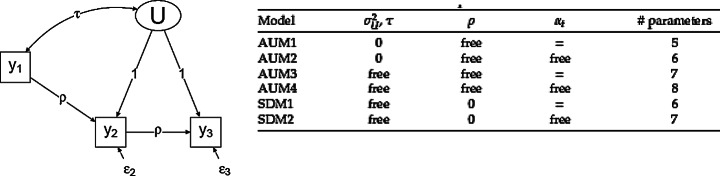
Rivalling models in SEM. (source: Vaisey & Kiley, 2021)

Varying the constraints to parameters in the model enables us to make a distinction between six structural models: four variations of the AUM model, and two variations of the SDM model (see Fig. 2). Figure 2 describes the different configurations of the SEM-model, that differ on the set of parameters that are fixed. Most notably, the dispositional models (SDM) fixate the effect of trust in observation 1 (Y1) on trust in observation 2 (Y2), as well as the effect of trust in observation 2 (Y2) on trust in observation 3 (Y3) to be 0, given the effect of the factor (U). By contrast, the socialization models (AUM) allow respondents to update their trust in observation 2 (Y2) by their trust in observation 1 (Y1), and their trust in observation 3 (Y3) by their trust observation 2 (Y2) with a non-zero effect, given factor (U). This crucial distinction differentiates the structural models of AUM and SDM in line with Fig. 1.

While we estimated all six models in Fig. 2 for each variable of interest, some models failed to converge successfully. Notably, models AUM3 and AUM4 were less likely to converge. Although this might imply some bias in favor of SDM models, we found little evidence that this affected our conclusions.

To assess the fit of the maximally six rivalling model specifications, we rely on two primary measures. First, BIC measures tell us which of the various models fit the data best. A lower BIC indicates a better model fit, taking account of the number of parameters that need to be estimated. If the difference in BIC between the best fitting AUM and SDM models is small, we conclude that the test is inconclusive. A rule of thumb reads that a difference in BIC of less than 2 constitutes a small difference, and thus weak evidence that one model is indisputably preferred over the other (cf. Raftery, 1995). We label a difference in BIC smaller than 1.5 as inconclusive, and mark the scarce cases where this difference is close to 2 as weak evidence (cf. Vaisey & Kiley, 2021: 88). Second, we report the RMSEA, which tells us whether the model fit is acceptable. The rule of thumb is that the RMSEA should be lower than 0.08. As model fit is key to distinguish between the rivalling models, we do not need to show the estimates of all individual parameters of the relationships within these models (cf. Vaisey & Kiley, 2021).

### Data

The analysis requires panel data encompassing a minimum of three sequential observations per respondent, covering both adolescents and adults, and incorporating measures of institutional trust. To meet these demands, we employ two data sets: the Dutch Adolescent Panel on Democratic Values (DAPDV; ADKS in Dutch) and the Longitudinal Internet studies for the Social Sciences (LISS). Both data sets are collected in the Netherlands, a country conventionally recognized for its high levels of trust in cross-national comparisons of democracies such as the World Values Survey and the European Social Survey (see Torcal, 2017).

DAPDV is a panel study of democratic values and attitudes among Dutch adolescents in secondary education collected by the University of Amsterdam between 2018 and 2024. Data collection was organized via two-stage sampling, in collaboration with (and generally at) the schools, and required active consent for participation by students, one parent, and the school. The first wave of DAPDV approached students at the average age of twelve, mere months after they were sorted into different secondary schools. Students were reapproached in subsequent years. After wave 4 (2022), students in prevocational education naturally dropped out of the sample, as they left secondary education. Hence, we rely on data of students between the ages of 12 and 16, collected between 2018 and 2022. The tables report students by the age at which they first participated (i.e., 12–13 when they started in wave 1, or 13–14 when they started in wave 2). The number of students in each wave is 2,390 (wave 1), 2,215 (wave 2), 1,219 (wave 3), and 1,624 (wave 4). Some schools and classes dropped out during the pandemic years; additionally, we experienced panel attrition as a result of students transferring to different classes or schools.

The LISS panel is a panel study in the Netherlands that has run since 2008, collected by Centerdata based on regularly refreshed samples by Statistics Netherlands. The sample comprises approximately 7,500 individuals of 16 years and older nested in 5,000 households, who are invited to participate in one or multiple questionnaires per month. The panel includes eight core questionnaires that are repeated annually. We rely on four waves that run in parallel with the DAPDV data as much as possible, i.e., the waves collected around January 2019, 2020, 2021, and 2022. Sample sizes per wave vary between 4,410 (wave 4) and 4,794 (wave 3). We break down the respondents in the LISS panel into five age groups, based on their age in 2019: 16–19 year olds, 20–29; 30–44; 45–64; and 65+.

The comparison between age groups benefits from relatively large sample sizes per group. Smaller sample sizes may bias estimates in favor of SDM.^3^ Our models are based on respondents who participated in all three waves. The analysis of the adolescents who first participated as 12–14 year olds in the DAPDV is based 1015 net respondents. The smallest group in the LISS panel are the 16–19 year olds from 2019 (194 respondents). The other groups are substantially larger: 20–29 year olds (517 respondents), 30–44 year olds (908), 45–64 year olds (1800) and 65+ (1441). Particularly the DAPDV thus suffers from missing values. Most of that is due to attrition of schools during the COVID years (2020–2022).^4^

Both data sets cover measures of trust in institutional authorities. Five of these trust objects are very similar, although DAPDV used simpler terms than the more common measures in LISS. This adjustment was made to accommodate the knowledge levels of young adolescents in different educational tracks. DAPDV asked respondents to what extent they agree (on a Likert scale) with the statement ‘I have a lot of trust in…’, followed by five institutional authorities as trust objects: [medical] doctors, judges, police officers, politicians (literal phrasing: people who work in politics), and the military. The LISS panel contains the question ‘Can you indicate, on a scale from 0 to 10, how much confidence you personally have in each of the following institutions?’.^5^ It covers seventeen trust objects, including five that align well with those in DAPDV: healthcare, the legal system, the police, politicians, and the military. The biggest difference between the two sets of measures is the number of answer categories. Yet, it is not evident that this difference will affect the relative model fit of the AUM and SDM.

The timing of data collection is a relevant background to the analysis. The first two waves of data were collected before the COVID pandemic hit the Netherlands. The third wave was collected in the first months of 2021, not only amidst a partial lockdown but also shortly before (LISS) and after (DAPDV) early parliamentary elections. The fourth wave (2022) was collected after the COVID crisis had ended and a new government coalition was in place. The third wave may therefore be exceptional. Particularly trust in politics shows considerable volatility during the 2019–2022 time span. Before the pandemic, political trust had been on the rise (Van der Meer & Van Erkel, 2024). This was followed by a substantial boost that is understood as a rally ‘round the flag during the initial months of pandemic itself in March and April 2020 (Van der Meer et al., 2023). In 2021, though, political trust showed a vast drop across the board.

We deal with this volatility in our analysis in two ways. First, the population-wide changes should not bias our analyses (Kiley & Vaisey, 2020: 480–481). Both the AUM and SDM allow for large changes in the population. In this regard, the main difference between the models is that SDM models expect such changes to revert to the baseline over time at the individual level. The four waves in our analysis do not show a major trend on most variables, and mainly volatility on political trust. Hence, there should not be a bias.

Second, we nevertheless check to what extent our analyses are sensitive to the inclusion or exclusion of waves 3 and 4. Our main analysis focuses on the first three waves that respondents participated in. Not all respondents participated in all waves. If we had focused exclusively on participants in all four waves, we would have lost a substantial number of respondents. We run robustness checks on respondents that only participated in waves 1–3 (taking out wave 4), and on respondents that only participated in waves 1, 2, and 4 (taking out wave 3). We draw similar conclusions from these alternate analyses.

Finally, a risk of panel data is the occurrence of panel effects: stable answer patterns that are not merely measured but actually induced by repeated participation in the survey. Fortunately, the LISS panel allows us to assess the extent to which our findings are sensitive to such panel effects. A second robustness check below shows that our conclusions are robust.

## Results

### Descriptive Analysis

**Fig. 3:**
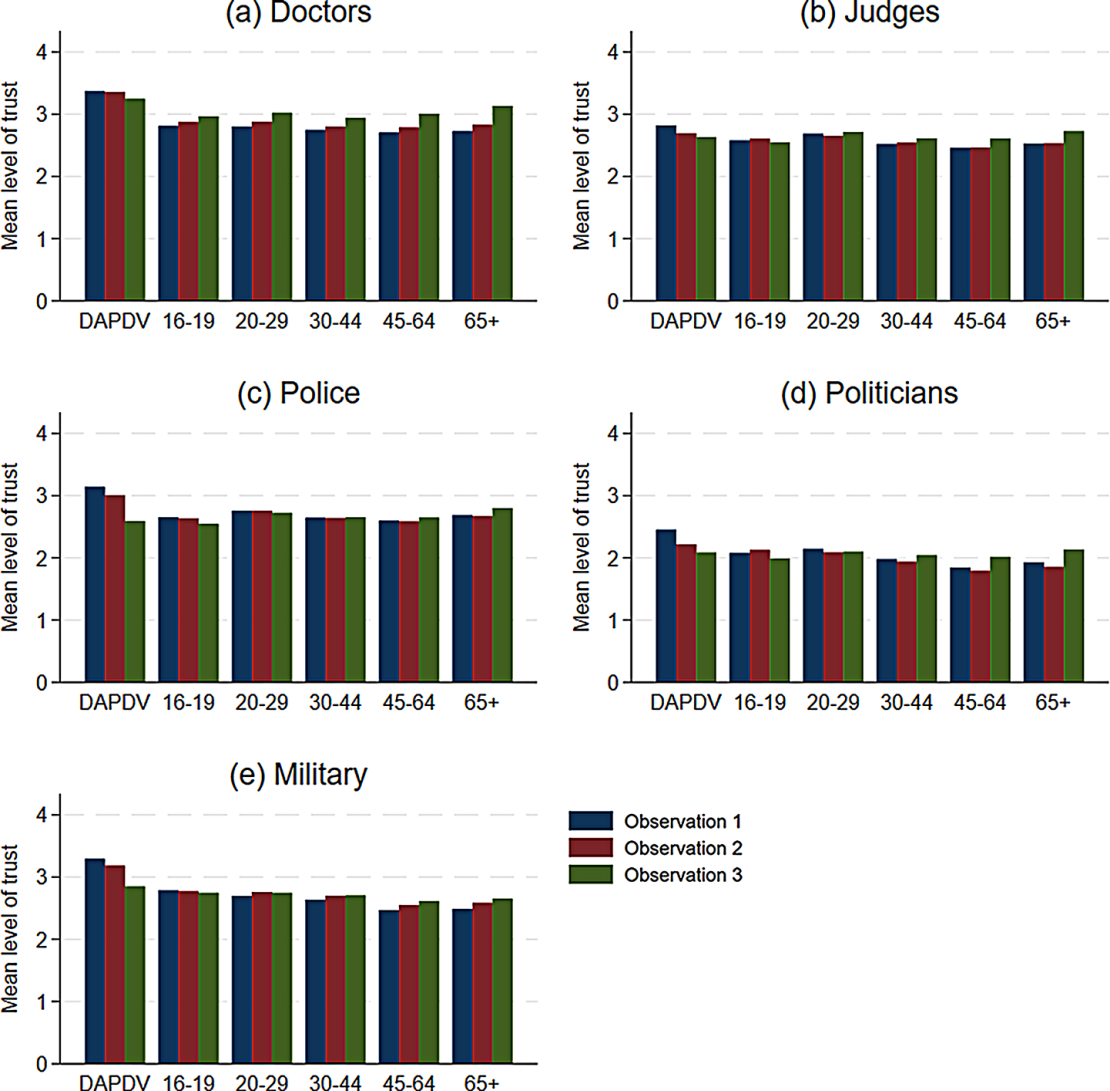
Mean level of trust across all five institutions by observation and age group (DAPDV scale)

Before proceeding with our formal test, we first explore patterns of stability and change in the aggregated data. Figure 3 displays the average level of trust across all three observations, segmented by age group. Across the majority of institutions, the average trust levels demonstrate a high degree of stability for all age groups in the LISS sample, but a constant decline among adolescents. Yet, there is one exception to this pattern: trust in doctors (Fig. 3a). Trust in doctors sees a consistent increase across all age groups in the LISS panel and stability among adolescents. This distinct pattern may be a result of the COVID-19 pandemic, considering that the increase is particularly visible among older respondents.

Table 1 summarizes these shifts in trust levels across observations. As the table shows, changes in trust over time are considerably larger and more persistent for the DAPDV sample. Additional descriptive figures confirm that, while complete stability across the three observed survey waves is the most common response pattern in all age groups, it is most prevalent among adults (see Appendix). However, stability does not form conclusive evidence of either the AUM or SDM. It merely indicates the absence of any external factors that have the capacity to influence trust – either lastingly (AUM) or temporarily (SDM). In contrast, the pattern of survey responses bouncing back and forth over time, which tends to be indicative of the SDM, is more commonly observed in adults. Yet, a formal test is required to determine whether the trust trajectories follow the AUM or SDM.

**Table 1:** Mean level of change between observations by age group (age at first wave)

Trust in	Sample 1 DAPDV 12–14			Sample 2 LISS 30–44		
		16–19	20–29		45–64	65+
Doctors	-0.06	**+ 0.05** [+ 0.21]	**+ 0.08** [+ 0.26]	**+ 0.07** [+ 0.26]	**+ 0.11** [+ 0.38]	**+ 0.16** [+ 0.52]
Judges	-0.11	**-0.00** [-0.05]	**+ 0.03** [+ 0.07]	**+ 0.04** [+ 0.14]	**+ 0.05** [+ 0.19]	**+ 0.07** [+ 0.25]
Politicians	-0.22	**-0.02** [-0.09]	**-0.02** [-0.04]	**+ 0.03** [+ 0.09]	**+ 0.09** [+ 0.22]	**+ 0.08** [+ 0.26]
Military	-0.22	**-0.04** [-0.02]	**+ 0.03** [+ 0.05]	**+ 0.02** [+ 0.10]	**+ 0.05** [+ 0.18]	**+ 0.06** [+ 0.22]
Police	-0.25	**-0.05** [-0.14]	**-0.01** [-0.02]	**+ 0.01** [+ 0.03]	**+ 0.02** [+ 0.06]	**+ 0.03** [+ 0.14]

Change in LISS data displayed in DAPDV scale. Original scale in square brackets.

### AUM vs. SDM

Table 2 shows the results of the tests of the rivalling SEM models. The findings are split by trust object (in the rows) and age groups (in the columns).

The first thing to stand out is the variation across age. Among young adolescents (12–14 year old in the DAPDV data set), the AUM dominates. Changes in trust tend to be driven by active updating from earlier waves rather than by an established disposition. We see evidence for the AUM among this age group for trust in doctors, politicians, the military, and the police. The SDM is only the better fit for trust in judges at this age group. Among late adolescents (16–19 years old in the LISS data set), we see much more evidence for the existence of settled dispositions. The SDM is the better fit for four of the five trust objects. Only trust in the military is suggestive of active updating. Finally, among adult age groups, the SDM model continuously dominates. We can safely conclude that there is a lot of evidence for stable dispositions among adults, even in the face of volatile trends of institutional trust.

All in all, we find support for our first hypothesis on the impressionable years, and reject the rivalling hypotheses that institutional trust always has a dispositional element (even at a young age) or that institutional trust never settles into a dispositional element (even at an older age).

**Table 2:** Preferred models, determined by BIC comparisons, by age group (age at first wave)

Trust in	Sample 1 DAPDV 12–14			Sample 2 LISS 30–44		
		16–19	20–29		45–64	65+
Doctors	AUM	SDM	SDM	SDM	SDM	SDM
Judges	SDM	SDM	SDM	SDM	SDM	SDM
Politicians	AUM	SDM	SDM	SDM	SDM	SDM
Military	AUM	AUM	SDM	SDM	SDM	SDM
Police	AUM	SDM	SDM	SDM	SDM	SDM

Second, we can check the model fit in terms of RMSEA (see Table 3).^6^ We find rather consistent evidence of a good model fit (RMSEA < 0.08) among young people. Interestingly, among older people, the model fit tends to decrease. Particularly among those who are older than 65 years old, model fit drops beyond conventionally acceptable levels. While the SDM is the best fitting model, even that best fitting model does not fit the data particularly well for older people’s trust in doctors (RMSEA = 0.19), judges (0.14), and politicians (0.11). This cannot be explained by issues such as sample size.

Across the rows, we can see that trust in doctors and the healthcare system (and to a lesser extent: trust in judges) tend to have a somewhat lower model fit than trust in other objects. These attitudes may be structured less well in line with the model specification. We can only speculate what could drive these differences. It cannot be due to the existence of non-attitudes per se: non-attitudes could be in line with the SDM, as random changes imply that trust rates tend to revert to the mean. One potential explanation could be that the nature of trust in these objects changed somewhat over the time span covered in this analysis. For instance, the pandemic of 2020 and 2021 shifted the public perspective on the healthcare system. It not only affected the salience of the institution, but also reshaped the very meaning of trusting or distrusting the healthcare system. That is, people were faced with mortality and infection, a rush for tests and treatments, and the increased circulation of false information regarding public health interventions (Beller et al., 2023).

**Table 3:** Model fit of the preferred models (RMSEA), by age group (age at first wave)

Trust in	Sample 1 DAPDV 12–14			Sample 2 LISS 30–44		
		16–19	20–29		45–64	65+
Doctors	0.05	0.08	0	0.10	0.07	0.19
Judges	0.06	0.08	0.06	0.02	0.08	0.14
Politicians	0	0.06	0	0.03	0.04	0.11
Military	0.05	0.06	0	0.05	0.05	0
Police	0	0.05	0.02	0	0.03	0.03

### Robustness check I: COVID

A first robustness check is centered around the unique and impactful event of the COVID-19 pandemic. Although the pandemic mainly affected levels of trust in political authorities (cf. Van der Meer et al., 2023), its widespread disruptive impact calls for an assessment of its effect on the analysis for all measures of institutional trust. We therefore re-estimated our models, once for waves 1, 2, and 3 (ending with the pandemic wave) in Table 4, and once for waves 1, 2, and 4 (skipping the pandemic wave to 2022, when concern with infection and vaccination had waned) in Table 5.

**Table 4:** Preferred models (including COVID pandemic year), determined by BIC comparisons, by age group (age at first wave)

Trust in	Sample 1 DAPDV 12–13			Sample 2 LISS 30–44		
		16–19	20–29		45–64	65+
Doctors	AUM	SDM	SDM	SDM	SDM	SDM
Judges	AUM	SDM	SDM	SDM	SDM	SDM
Politicians	AUM	SDM	SDM	SDM	SDM	SDM
Military	AUM ^***a***^	AUM	SDM	SDM	SDM	SDM
Police	SDM ^***b***^	SDM	SDM	SDM	SDM	SDM

Model covers waves 1 (2018/2019), 2 (2019/2020) and 3 (2021)

^a^ AUM2 had a lower BIC than SDM, but barely within a margin of 2 points; AUM3 would not converge

^b^ Models AUM3 and AUM4 would not converge

**Table 5:** Preferred models (excluding COVID pandemic year), determined by BIC comparisons, by age group (age at first wave)

Trust in	Sample 1 DAPDV 12–13			Sample 2 LISS 30–44		
		16–19	20–29		45–64	65+
Doctors	AUM	AUM	SDM	SDM	SDM	SDM
Judges	SDM	SDM	SDM	SDM	SDM	SDM
Politicians	SDM ^***a***^	*inconclusive*	SDM	SDM	SDM	SDM
Military	AUM	AUM ^b^	SDM	SDM	SDM	SDM
Police	AUM	*inconclusive*	SDM	SDM	SDM	SDM

Model covers waves 1 (2018/2019), 2 (2019/2020) and 4 (2022)

^a^ Models AUM3 and AUM4 would not converge

^b^ AUM2 had a lower BIC than SDM, but barely within a margin of 2 points; AUM3 would not converge

When we zoom out, the overall conclusions seem to be robust to the in- or exclusion of 2021. Both Tables 4 and 5 show that the SDM (i.e., dispositions) prevails among adults, that the AUM (i.e., socialization) offers the best description among young adolescents, and that late adolescents fall somewhat in between. In other words, regardless of the pandemic we continue to find support for the impressionable years hypothesis over the two rivalling hypotheses.

Yet, upon closer inspection, there is some variation across the different analyses. Among young adolescents we only find consistent support for AUM for trust in doctors and trust in the military. For this age group, however, trust in the police showed evidence for SDM over AUM in the first robustness check (including the pandemic year), and trust in politicians in the second (excluding the pandemic year). Among late adolescents and young adults (16–19 years old), evidence for SDM over AUM is less conclusive when we exclude the pandemic year of 2021. When we do that, we find evidence for updating on trust in doctors, and reach inconclusive results on trust in politicians and the police. Finally, among adults (20+) we find consistent support for SDM over AUM in the main analyses as well as the robustness checks.

### Robustness Check II: Panel Effects (LISS)

A second risk is that of panel effects in the LISS panel. Because members of the LISS panel tend to answer questionnaires with great regularity, their repeated participation might induce stable dispositions or stable answer patterns. In other words, longstanding LISS panel members might be socialized towards SDM. For that purpose, we selected a subsample of LISS panel respondents that have only been selected to participate in more recent years and should have been less susceptible to such panel effects. The two most recent refreshment samples were collected in 2016 and in 2019/2020. The panel data in this paper contain 823 respondents from these two samples. We have been able to estimate AUMs and SDMs for all age groups. However, the outcomes for the youngest age group (16–19 years old) tended to be inconclusive, corresponding to the very small sample size: we ended up with merely 28 respondents within this age group.

Among the other groups, we find consistent support for SDM over AUM, mirroring our findings in the main analysis. To conclude, there is no indication that the prevalence of the SDM among adults is driven by panel effects.

### Exploratory Analysis: Interest and Discussion (DAPDV)

Finally, we assess to what extent evidence for the impressionable years hypothesis hinges on young adolescents’ socio-political interest and engagement in discussions on social and political issues. SDM cannot really distinguish between dispositions and non-attitudes. To shed light on this distinction, we retest the models on the most and least interested/engaged respondents (each group represents approximately a third of the sample). Table 6 shows that the relatively interested respondents (who score an average higher than 3 on a 0 to 4 scale) show more evidence for SDM, whereas the relatively disinterested respondents (lower than 2 on that scale) show predominant evidence for AUM. This suggests that among interested adolescents, dispositions to trust are more likely to settle early on. It also implies that the support for SDM are not due to non-attitudes.

The latter conclusion is further supported when we distinguish between those who are relatively more and less engaged in political discussions – an engagement that, to be fair, is rather marginal among both groups. DAPDV asked about engagement in talks about social and political issues with parents, teachers, and friends. The most engaged respondents (on average more than several times per year) mainly show evidence in favor of AUM, although there is also evidence for SDM for trust in judges. This is very similar to the main analyses. Among those who are even less engaged in these discussions (on average less than several times per year), there is no evidence for SDM at all.

**Table 6:** Preferred models, determined by BIC comparisons, by socio-political interest and engagement in socio-political discussions (DAPDV sample only)

Trust in	High interest	Low interest	Somewhat frequent discussion	Very little discussion
Doctors	AUM	AUM	*inconclusive*	AUM
Judges	SDM	*inconclusive*	SDM	AUM
Politicians	SDM	AUM	AUM	AUM
Military	AUM	AUM	AUM	*inconclusive*
Police	SDM	AUM	AUM	AUM

## Conclusion

A longstanding argument in the field of institutional trust reads that trust is the outcome of a process of socialization. This approach suggests that institutional trust may be understood as a set disposition that is shaped during one’s impressionable years (i.e., adolescence and pre-adulthood) and no longer systematically updated during an iterative process afterwards. Consequently, this disposition forms a baseline around which trust judgments tend to vary. To date, the literature has offered indirect evidence in favour of this hypothesis focusing on the size rather than the structure of changes over time (e.g., Devine & Valgarðsson, 2024). The dispositional element of institutional trust may find its origins at a relatively early age (Claes et al., 2012; Hooghe et al., 2015). Yet, this process of socialization to (dis)trust has not been studied directly.

For that purpose, we employed two panel data sets in the Netherlands that measure institutional trust annually: the LISS panel (covering the adult population) and the Dutch Adolescent Panel on Democratic Values (covering students in secondary education from age 12). Structural equation modelling allowed us to test two rivalling models (Kiley & Vaisey, 2020; Vaisey & Kiley, 2021): the active updating model (in which attitudinal baselines are updated durably over time) and the settled dispositions model (in which attitudes remain relatively stable over time).

Our findings provide firm evidence for socialization among the youngest people (who still update their trust attitudes) as well as for disposition among older cohorts (who have a more stable base level of trust). Whereas the idea of socialization (i.e., the AUM) tends to fit the panel data best among adolescents (12–16 years old), dispositions (i.e., the SDM) tend to have stabilized among adults (20 years and older). Among young adults (16–19 years old) evidence is somewhat more mixed. This suggests not only that both socialization and dispositions matter, but also that these trust attitudes tend to stabilize at a relatively young age.

What do these findings mean? It is unlikely that the small and relatively meaningless short-term changes in institutional trust (see also Devine & Valgarðsson, 2024) are due to two alternative explanations. The first reads that they are the outcome of habituation with survey questions: young participants learn to answer survey questions consistently, whereas old panel members are habituated. Our robustness checks on new survey panel members suggest that this is unlikely to explain our findings. The second alternative explanation reads that the dispositional model may reflect non-attitudes. It is true that the method used in this paper cannot distinguish dispositions from non-attitudes. Nevertheless, non-attitudes are unlikely. Non-attitudes cannot explain why individual variation occurs on such a small bandwidth (Devine & Valgarðsson, 2024), while macro-micro effects tend to be so consistent across the literature (e.g., Hetherington, 1998; Bowler & Karp, 2004; Van der Meer & Van Erkel, 2024).

The dispositional model does not need to be at odds with the evaluative model. Citizens in open democratic societies with low levels of partisan polarization may be socialized to evaluate. They are likely to respond to external stimuli such as institutional performance, but do so around their baseline of trust. In fact, the dispositional model that we tested in this paper is open to longitudinal changes in institutional trust that in the long run tend to revert to the mean. This is what we observe, specifically in recent political trust research. Political trust tends to be very reactive to political performance in the short term (cf. Ouattara et al., 2023). Yet, in the long run political trust generally tends to revert to its baseline, that is set by states’ institutional performance (Van der Meer & Van Erkel, 2024) and by citizens’ own dispositions. Ultimately, the integration of the dispositional and evaluative models may help explain the conundrum in unidimensional scaling research (such as factor and hierarchical analyses) that institutional trust is simultaneously an object-specific evaluation and a non-specific trait (cf. Aruqaj, 2023; Van der Meer & Ouattara, 2019).

The support we find for the dispositional element of institutional trust and the impressionable years hypothesis has wider theoretical and methodological implications. First, it supports the recent push in the literature to distinguish between short-term and long-term drivers and consequences of institutional trust, not only theoretically but also methodologically, by separating stable and dynamic features of trust. We should further our search into movement around more or less stable dispositions at different stages in life. The extent to which political trust is stable or volatile, as well as the implications thereof, is a relevant focus in itself. Second, to the extent that adolescents and young adults develop dispositions, we need to push for a better understanding of these socialization processes, not only by focusing on the influence of traditional socializing agencies such as parents and schools, but also by assessing the impact of short-term and long-term institutional performance. Third, the trust literature needs to focus on age and cohort not only as standard control variables, but also as methodological leverage to test theories of socialization and evaluation. To the extent that dispositions take shape and crystallize at a young age, young people are more likely to develop between-person variation than old people. To the extent that trust is an object-specific evaluation, old people are more likely to show within-person variation. Yet, concurrently, younger cohorts may be more likely to be socialized to evaluate as critical citizens than older generations (cf. Norris, 1999; Dalton, 2004). It seems unlikely that the prevalence of the AUM among adolescents can be attributed to the impact of transformative life events (cf. Lersch, 2023). While the COVID-19 pandemic was a major transformative life event for all age groups during the observed period, the SDM appears to better explain attitude development among adults and young adults than among adolescents. Nevertheless, it would be good to retest the models during less volatile years.

We have been able to uncover evidence of both adaptation and disposition in a country that is conducive to such analysis, due to its low to moderate levels of economic inequality, social segregation, and political polarization. We can only speculate about the relative importance of both mechanisms in countries other than the Netherlands. First, high levels of polarization – particularly in combination with segregation - might make it more difficult to identify the socialization mechanism, as high levels of partisanship may overwhelm identity formation within communities. Second, the stabilization of dispositions in the Netherlands seems to coincide with legal and economic adulthood (political rights, legal rights, entrance to the labour and/or housing market). Possibly, this stabilization might occur at a younger age in countries where legal or economic adulthood occurs (*de jure* or *de facto*) at a younger age.

All in all, after the institutional trust literature put the evaluative approach to institutional trust under increased scrutiny, this paper shows that dispositions play an important role among adults and that they are shaped during late adolescence and young adulthood. It is remarkable that we found such consistent evidence for dispositions during a time of great political upheaval, including the COVID-19 pandemic and the subsequent lockdown, a government breakdown, and various political scandals. Nevertheless, it is worthwhile to extend the test, to cover multiple governmental periods with differing government compositions, preferably across multiple countries. That would allow us to reach into the partisan nature of disposition to distrust societal institutions.

## Footnotes

1 Evidence for this hypothesis is mixed. At the micro-level, Ouattara et al. (2023) find little evidence. At the macro-level, Van der Meer and Van Erkel (2024) find substantial evidence.

2 Note that this approach is not inherently incompatible with the trust-as-disposition model. The primary concern is not *whether* trust is either stable or volatile, but *to what extent* it consists of a dispositional element (see Devine & Valgarðsson, 2024). For instance, a more nuanced account of the nature of institutional trust might suggest that trust forms a settled baseline after the impressionable years, but that individuals’ trust levels vary around this baseline on the basis of evaluations over time.

3 Interestingly, this suggests that our findings are conservative.

4 Unfortunately, there is no optimal solution to deal with this issue. In principle, listwise deletion takes away statistical power and assumes random missingness. However, missing value imputation is a risk, when missingness is mainly due to respondents missing out one (of already rather few) whole waves. This is particularly problematic in our study, because most dropout took place at the school and class levels, because of the severe limitations for data collection under social segregation and (partial) lockdowns during the COVID pandemic. We did not find evident patterns in the attrition of classes and schools. All in all, under these circumstances, missing value imputation is not likely to benefit statistical power but risks adding randomness to a study on attitude formation.

5 Note that the official LISS panel documentation uses the translation ‘confidence’ instead of ‘trust’. Both refer to the exact same word in Dutch: ‘vertrouwen in’.

6 Several model fit parameters are estimated to be zero; yet, the upper bound confidence intervals are not.
